# Poly[μ-(5,5′-diazenediylditetra­zolido)-dicaesium]

**DOI:** 10.1107/S1600536811008312

**Published:** 2011-03-15

**Authors:** Yan Meng

**Affiliations:** a School of Environmental Engineering, Chang’an University, South Second Cycle Road 368#, Xi’an 710064, Shaanxi, People’s Republic of China

## Abstract

The asymmetric unit of the title compound, [Cs_2_(C_2_N_10_)]_*n*_, comprises a Cs^+^ cation, and one-half of a 5,5′-diazenediylditetra­zolide anion. The Cs^+^ cation is six-coordinated by N atoms from six 5,5′-diazenediylditetra­zolide ligands. Each 5,5′-diazenediylditetra­zolide ligand is surrounded by 12 Cs^+^ cations, coordinating through ten N atoms. The Cs^+^ cations are arranged in a chain along the *a*-axis direction with a Cs⋯Cs separation of 4.4393 (10) Å. Such coordination leads to the formation of the three-dimensional framework.

## Related literature

For applications of 5,5′-diazenediylditetra­zolide salts, see: Hammerl *et al.* (2001 ▶). For the synthesis of sodium 5,5′-diazenediylditetra­zolide, see: Thiele (1892 ▶). For the synthesis and characterization of alkali and alkaline earth metal salts of 5,5′-diazenediylditetra­zolide, see: Hammerl *et al.* (2002 ▶); Steinhauser *et al.* (2009 ▶). For Cs—N bond lengths, see: Ebespächer *et al.* (2009 ▶). 
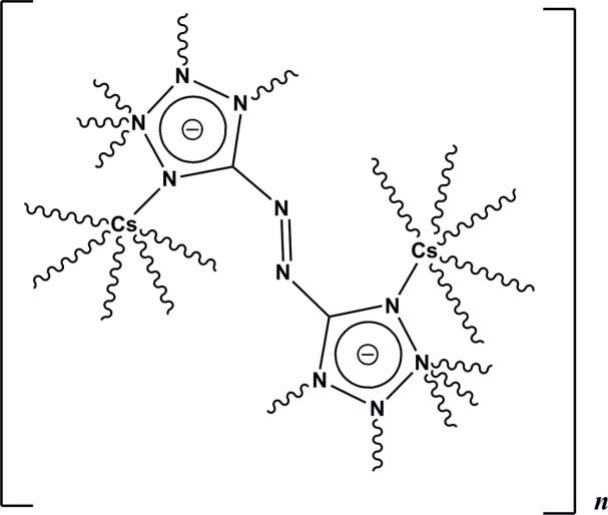


## Experimental

### 

#### Crystal data

[Cs_2_(C_2_N_10_)]
*M* *_r_* = 429.94
Monoclinic, 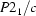

*a* = 4.4393 (9) Å
*b* = 8.7151 (17) Å
*c* = 11.860 (2) Å
β = 93.83 (3)°
*V* = 457.82 (16) Å^3^
*Z* = 2
Mo *K*α radiation
μ = 7.94 mm^−1^
*T* = 293 K
0.42 × 0.26 × 0.07 mm

#### Data collection

Bruker SMART CCD diffractometer
Absorption correction: multi-scan (*SADABS*; Bruker, 2007 ▶) *T*_min_ = 0.135, *T*_max_ = 0.606
4146 measured reflections
842 independent reflections
747 reflections with *I* > 2σ(*I*)
*R*_int_ = 0.047

#### Refinement

*R*[*F* ^2^ > 2σ(*F* ^2^)] = 0.031
*wR*(*F* ^2^) = 0.082
*S* = 1.15
842 reflections
64 parameters
Δρ_max_ = 1.36 e Å^−3^
Δρ_min_ = −1.47 e Å^−3^

### 

Data collection: *SMART* (Bruker, 2007 ▶); cell refinement: *SAINT* (Bruker, 2007 ▶); data reduction: *SAINT*; program(s) used to solve structure: *SHELXTL* (Sheldrick, 2008 ▶); program(s) used to refine structure: *SHELXTL*; molecular graphics: *SHELXTL*; software used to prepare material for publication: *publCIF* (Westrip, 2010 ▶).

## Supplementary Material

Crystal structure: contains datablocks I, global. DOI: 10.1107/S1600536811008312/nk2085sup1.cif

Structure factors: contains datablocks I. DOI: 10.1107/S1600536811008312/nk2085Isup2.hkl

Additional supplementary materials:  crystallographic information; 3D view; checkCIF report

## Figures and Tables

**Fig. 1.**
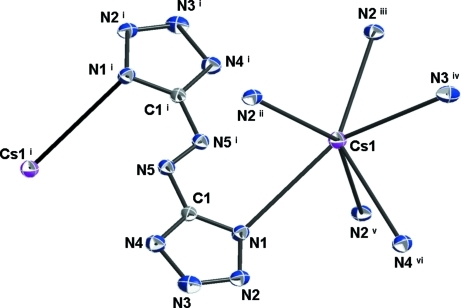
The structure of (I), with 30% probability displacement ellipsoids. Symmetry code: (i) – x, – y + 1, – z + 1; (ii) x + 1, y, z; (iii) – x, y – 1/2, – z + 1/2; (iv) x + 1, – y + 1/2, z – 1/2; (v) – x – 1, y + 1/2, – z + 1/2;(vi) x, – y + 1/2, z – 1/2.

**Fig. 2.**
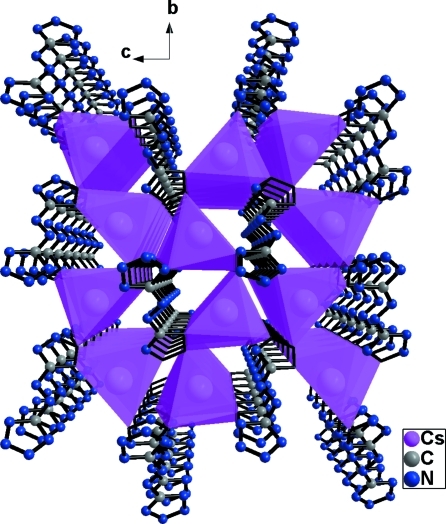
The three-dimensional framework of (I).

## supplementary crystallographic information

### Comment

Salts of 5,5'-diazenediylditetrazolide are powerful gas generation agents and can be used in gas generators for airbags and fire extinguishing systems (Hammerl *et al.*, 2001). Thiele first prepared sodium 5,5'-diazenediylditetrazolide (Thiele, 1892), which is usually used as the starting material for other 5,5'-diazenediylditetrazolide compounds. Up to now, although many alkali-and alkaline earth metal salts of 5,5'-diazenediylditetrazolide have been prepared (Hammerl *et al.*, 2002; Steinhauser *et al.*,2009), more work still needs to be done. In this paper, we report the crystal structure of the title compound, (I), a new Cs complex obtained by the reaction of sodium 5,5'-diazenediylditetrazolide and CsCl in water.

The asymmetric unit of the title compound comprises a Cs^+^ cation, and a half of 5,5'-diazenediylditetrazolide anion. The central cation is coordinated to six N atoms from six 5,5'-diazenediylditetrazolide ligands (Fig. 1) with the Cs—N distances ranging from 3.225 (6) Å to 3.341 (5) Å, which are well within the range reported in the literature (Ebespächer *et al.*, 2009). The atom N2 from the tetrazole rings acts as *µ*_3_-bridge. Thus, each 5,5'-diazenediylditetrazolide anion links twelve Cs^+^ cations through ten nitrogen atoms. The Cs^+^ cations are arranged in a one-dimensional chain along the *a*-axis direction with the Cs^+^ ··· Cs^+^ separation of 4.4393 (10) Å. Such linking mode leads to the formation of the three-dimensional framework of the title compound (Fig. 2).

### Experimental

To a solution of sodium 5,5'-diazenediylditetrazolide in 20 ml bidistilled water, a solution of CsCl was added dropwise at room temperature. After stirring for 30 minutes a yellow solution was obtained after filtration. The filtrate was then set aside for crystallization at room temperature. Three weeks later, yellow block crystals of the title compound suitable for X-ray determination were isolated.

### Refinement

All atoms were refined anisotropically. The maximum residual electron density of 1.36 e Å^-3^ is located 1.11 Å from Cs1 and the minimum density of -1.46 e Å^-3^ lies 0.83 Å from Cs1.

### Crystal data

**Table:**

[Cs_2_(C_2_N_10_)]	*F*(000) = 384
*M**_r_* = 429.94	*D*_x_ = 3.119 Mg m^−^^3^
Monoclinic, *P*2_1_/*c*	Mo *K*α radiation, λ = 0.71073 Å
Hall symbol: -P 2ybc	Cell parameters from 1601 reflections
*a* = 4.4393 (9) Å	θ = 3.4–25.4°
*b* = 8.7151 (17) Å	µ = 7.94 mm^−^^1^
*c* = 11.860 (2) Å	*T* = 293 K
β = 93.83 (3)°	Block, yellow
*V* = 457.82 (16) Å^3^	0.42 × 0.26 × 0.07 mm
*Z* = 2	

### Data collection

**Table:**

Bruker SMART CCD diffractometer	842 independent reflections
Radiation source: fine-focus sealed tube	747 reflections with *I* > 2σ(*I*)
graphite	*R*_int_ = 0.047
φ and ω scans	θ_max_ = 25.3°, θ_min_ = 3.4°
Absorption correction: multi-scan (*SADABS*; Bruker, 2007)	*h* = −5→4
*T*_min_ = 0.135, *T*_max_ = 0.606	*k* = −10→10
4146 measured reflections	*l* = −12→14

### Refinement

**Table:**

Refinement on *F*^2^	0 restraints
Least-squares matrix: full	Primary atom site location: structure-invariant direct methods
*R*[*F*^2^ > 2σ(*F*^2^)] = 0.031	Secondary atom site location: difference Fourier map
*wR*(*F*^2^) = 0.082	*w* = 1/[σ^2^(*F*_o_^2^) + (0.0385*P*)^2^ + 0.5977*P*] where *P* = (*F*_o_^2^ + 2*F*_c_^2^)/3
*S* = 1.15	(Δ/σ)_max_ < 0.001
842 reflections	Δρ_max_ = 1.36 e Å^−^^3^
64 parameters	Δρ_min_ = −1.46 e Å^−^^3^

### Special details

**Table:**

Geometry. All e.s.d.'s (except the e.s.d. in the dihedral angle between two l.s. planes) are estimated using the full covariance matrix. The cell e.s.d.'s are taken into account individually in the estimation of e.s.d.'s in distances, angles and torsion angles; correlations between e.s.d.'s in cell parameters are only used when they are defined by crystal symmetry. An approximate (isotropic) treatment of cell e.s.d.'s is used for estimating e.s.d.'s involving l.s. planes.
Refinement. Refinement of *F*^2^ against ALL reflections. The weighted *R*-factor *wR* and goodness of fit *S* are based on *F*^2^, conventional *R*-factors *R* are based on *F*, with *F* set to zero for negative *F*^2^. The threshold expression of *F*^2^ > σ(*F*^2^) is used only for calculating *R*-factors(gt) *etc*. and is not relevant to the choice of reflections for refinement. *R*-factors based on *F*^2^ are statistically about twice as large as those based on *F*, and *R*- factors based on ALL data will be even larger.

### Fractional atomic coordinates and isotropic or equivalent isotropic displacement parameters (Å^2^)

**Table:**

	*x*	*y*	*z*	*U*_iso_*/*U*_eq_	
Cs1	0.08959 (8)	0.47092 (5)	0.19017 (3)	0.0345 (2)	
C1	−0.2743 (12)	0.3536 (7)	0.4829 (5)	0.0246 (13)	
N1	−0.3813 (11)	0.3517 (6)	0.3756 (4)	0.0320 (12)	
N2	−0.5901 (11)	0.2386 (6)	0.3729 (5)	0.0346 (13)	
N3	−0.6023 (13)	0.1805 (6)	0.4744 (5)	0.0389 (14)	
N4	−0.4029 (13)	0.2515 (7)	0.5477 (5)	0.0379 (14)	
N5	−0.0545 (11)	0.4536 (6)	0.5334 (5)	0.0293 (12)	

### Atomic displacement parameters (Å^2^)

**Table:**

	*U*^11^	*U*^22^	*U*^33^	*U*^12^	*U*^13^	*U*^23^
Cs1	0.0288 (3)	0.0391 (3)	0.0354 (3)	−0.00007 (16)	0.0014 (2)	0.00540 (17)
C1	0.022 (3)	0.026 (3)	0.026 (3)	0.005 (3)	0.001 (2)	−0.001 (3)
N1	0.028 (3)	0.035 (3)	0.032 (3)	−0.004 (2)	−0.001 (2)	−0.007 (3)
N2	0.026 (3)	0.034 (3)	0.043 (3)	−0.002 (2)	0.001 (2)	−0.009 (3)
N3	0.031 (3)	0.029 (3)	0.057 (4)	0.003 (2)	0.004 (3)	0.008 (3)
N4	0.031 (3)	0.040 (3)	0.042 (3)	0.001 (3)	0.001 (3)	0.009 (3)
N5	0.025 (3)	0.032 (3)	0.031 (3)	0.002 (2)	0.003 (2)	−0.003 (2)

### Geometric parameters (Å, °)

**Table:**

Cs1—N2^i^	3.225 (6)	N1—N2	1.352 (7)
Cs1—N3^ii^	3.260 (6)	N2—N3	1.311 (8)
Cs1—N2^iii^	3.270 (5)	N2—Cs1^vi^	3.225 (6)
Cs1—N4^iv^	3.301 (6)	N2—Cs1^vii^	3.270 (5)
Cs1—N1	3.301 (5)	N2—Cs1^viii^	3.341 (5)
Cs1—N2^v^	3.341 (5)	N3—N4	1.349 (8)
C1—N1	1.329 (7)	N3—Cs1^ix^	3.260 (6)
C1—N4	1.329 (8)	N4—Cs1^x^	3.301 (6)
C1—N5	1.411 (8)	N5—N5^xi^	1.252 (10)
			
N2^i^—Cs1—N3^ii^	94.82 (14)	N4—C1—N5	118.7 (5)
N2^i^—Cs1—N2^iii^	149.65 (5)	C1—N1—N2	103.4 (5)
N3^ii^—Cs1—N2^iii^	115.02 (14)	C1—N1—Cs1	115.6 (4)
N3^ii^—Cs1—N1^i^	94.53 (14)	N2—N1—Cs1	132.6 (4)
N2^iii^—Cs1—N1^i^	145.48 (14)	N3—N2—N1	109.3 (5)
N2^i^—Cs1—N4^iv^	102.85 (14)	N3—N2—Cs1^vi^	144.7 (4)
N3^ii^—Cs1—N4^iv^	70.05 (14)	N1—N2—Cs1^vi^	80.2 (3)
N2^iii^—Cs1—N4^iv^	83.47 (14)	N3—N2—Cs1^vii^	82.3 (4)
N2^i^—Cs1—N1	68.00 (13)	N1—N2—Cs1^vii^	168.2 (4)
N3^ii^—Cs1—N1	135.48 (14)	N3—N2—Cs1^viii^	90.3 (4)
N2^iii^—Cs1—N1	85.81 (13)	N1—N2—Cs1^viii^	92.8 (3)
N4^iv^—Cs1—N1	74.28 (13)	N2—N3—N4	110.4 (5)
N2^i^—Cs1—N2^v^	108.58 (7)	N2—N3—Cs1^ix^	157.3 (4)
N3^ii^—Cs1—N2^v^	77.69 (14)	N4—N3—Cs1^ix^	88.4 (4)
N2^iii^—Cs1—N2^v^	84.36 (13)	C1—N4—N3	102.9 (5)
N4^iv^—Cs1—N2^v^	136.31 (13)	C1—N4—Cs1^x^	113.2 (4)
N1—Cs1—N2^v^	146.00 (14)	N3—N4—Cs1^x^	116.4 (4)
N1—C1—N4	113.9 (5)	N5^xi^—N5—C1	114.6 (7)
N1—C1—N5	127.4 (6)		

Symmetry codes: (i) *x*+1, *y*, *z*; (ii) *x*+1, −*y*+1/2, *z*−1/2; (iii) −*x*−1, *y*+1/2, −*z*+1/2; (iv) *x*, −*y*+1/2, *z*−1/2; (v) −*x*, *y*+1/2, −*z*+1/2; (vi) *x*−1, *y*, *z*; (vii) −*x*−1, *y*−1/2, −*z*+1/2; (viii) −*x*, *y*−1/2, −*z*+1/2; (ix) *x*−1, −*y*+1/2, *z*+1/2; (x) *x*, −*y*+1/2, *z*+1/2; (xi) −*x*, −*y*+1, −*z*+1.
